# BV associated bacteria specifically BVAB 1 and BVAB 3 as biomarkers for HPV risk and progression of cervical neoplasia

**DOI:** 10.1155/2022/9562937

**Published:** 2022-08-13

**Authors:** Kavitha Naidoo, Nathlee Abbai, Partson Tinarwo, Motshedisi Sebitloane

**Affiliations:** ^1^University of KwaZulu-Natal, Department of Obstetrics and Gynaecology, School of Clinical Medicine, College of Health Sciences, South Africa; ^2^South African Medical Research Council-HIV and Other Infectious Diseases Research Unit, South Africa; ^3^University of KwaZulu-Natal, School of Clinical Medicine Laboratory, College of Health Sciences, South Africa; ^4^University of KwaZulu-Natal, Department of Biostatistics, College of Health Sciences, South Africa

## Abstract

**Background:**

Bacterial vaginosis (BV) is associated with high-risk HPV (hrHPV) genotypes. There is a proposed bidirectional relationship between hrHPV and vaginal microbial diversity. This study investigated the association between BV associated bacteria in women co-infected with Human immunodeficiency virus (HIV) and hrHPV.

**Methods:**

Stored cervical cytobrush samples were used for real time PCR detection of eight BV associated bacteria. Analysis of BV bacteria detected against HPV infection, socio-demographics and HIV data were conducted in R Statistical computing software of the R Core Team, 2020, version 3.6.3.

**Results:**

A total of 190 samples were analysed. *A. vaginae* (p <0.001) BVAB 1 (p <0.001), BVAB 2 (p =0.428), BVAB 3 (p <0.001), *Lactobacillus* species (p =0.016) and *S. sanguinegens* (p =0.007) were associated with prevalent hrHPV. Increasing CIN severity was independently associated with detection of BVAB 1 OR 1.51(95% CI: 0.42-5.55), BVAB 3 OR 2.72(95% CI:0.90-8.55) and *S. sanguinegens* OR 1.02(95% CI:0.37-2.80). All HPV genotypes/groups, gravida <2, *A. vaginae* (p =0.002) and BVAB 1 (p =0.026) were significantly associated with HPV persistence. BVAB 3, p =0.010 and HPV 16 were significantly associated with HPV reinfection.

**Conclusion:**

There is a significant association of *A. vaginae*, BVAB 1, BVAB 3, *S. sanguinegens* and *Lactobacillus* spp to prevalent hrHPV. BVAB 1, BVAB 3 and *S. sanguinegens* had an increased odds for increasing CIN severity. *A vaginae*, BVAB 1, gravida and all the HPV genotypes/groups were significantly associated with HPV persistence. Only BVAB 3 and HPV 16 were significantly associated with hrHPV reinfection at 1 year review. BVAB 1 and BVAB 3 are possible biomarkers for HPV infection and CIN progression.

## 1. Introduction

In South Africa, the vaginal microbiome of women of black ethnicity displayed a non-classical representation of a ‘normal' vaginal microbiome. While dominance of *Lactobacillus* species is considered the standard of vaginal health, South African women who were HIV negative and reported no symptoms of bacterial vaginosis (BV) were found to have a vaginal microbiome that was diverse in bacterial populations with a low *Lactobacillus* presence [1]. The representation of the vaginal microbiome found in South African women are correlated with a high vaginal pH and inflammatory cells which are associated with an increased risk of acquiring sexually transmitted infections (STIs) and other infections such as Human Papilloma virus (HPV) [2, 3].

Dysbiosis is an imbalance or change in the microbial vaginal environment. Women presenting with bacterial vaginosis have an elevated vaginal pH [4]. This is the result of dysbiosis and presence of a vaginal environment with high microbial diversity, which comprises of a large number of anaerobic bacteria [5]. It is likely that metabolic products produced by these bacteria results in the elevated pH observed during BV infection. Clarke et al. (2012) reported the association of HPV and vaginal pH. Elevated pH increased the risk of acquisition of multiple HPV genotypes and presentation of low-grade squamous intraepithelial lesions (LSIL) [2]. Additionally, bacterial vaginosis results in the increased shedding of vaginal epithelial cells, causing weakening of the epithelial barrier function. This increases the susceptibility to infection by invading microbes and viruses including sexually transmitted disease pathogens [6]. The pathogenesis of HPV is correlated with keratinocyte differentiation with the initial entry into the cell via injuries to the epithelium [7] HPV has an affinity for the basal layer and entry requires active division [8] Specific vaginal bacteria are able to evoke a proinflammatory response through the increased expression of membrane associated mucins, leading to a disruption of the immune barrier, thus exposing the basal cells [9].

In a study by Onywera et al. (2019), black South African women who were positive for high-risk HPV (hrHPV) genotypes had an increased abundance of *Aerococcaceae*, *Pseudomonadaceae* and *Bifidobacteriaceae*. The authors also found an increase in abundance of other BV associated bacteria namely *Gardnerella* and *Sneathea* spp. The population comprised of HIV negative women with a sample size of 87 [10]. In Polish women attending a cervical screening clinic, three *Lactobacillus* species were found to be dominant in healthy (HPV negative) women, which were absent in HPV positive women at high risk for high-grade squamous intraepithelial lesions (HSIL) [11]. In a group of HIV positive women enrolled from Johannesburg, South Africa, dysbiosis of the vaginal microbiome was significant in women who had a histology of cervical intraepithelial neoplasia grade 2 or higher (CIN2+) at least once during the study [12]. Another study also found that HPV or BV and HPV with BV were associated with an increased incidence of CIN or cervical cancer [13].

BV is associated with hrHPV genotypes and there is a proposed bidirectional relationship between hrHPV and vaginal microbial diversity [12]. Using molecular methods, we aimed to identify the association between specific BV associated bacteria within a cohort of HIV positive women co-infected with hrHPV. Furthermore, we elucidate the role of these bacteria in HPV persistence, reinfection and cervical neoplasia in this group of South African women on HAART.

## 2. Methods

### 2.1. Ethics Statement

The study was approved by the Biomedical Research Ethics Committee (BREC) of the University of KwaZulu-Natal, (BREC/00002905/2021). This study utilised stored samples from a larger Cervical Cancer Screening and Treatment Algorithm study (CESTA) with BREC approval BFC363/18 for which written informed consent was received for use of stored samples for future research.

### 2.2. Study design and population

This was a retrospective sub-study of the CESTA study which enrolled HIV positive women between the ages of 25-54 years. A total of 400 women were enrolled. One hundred and 90 samples from women who had completed the study were available for analysis. HIV positive women attending the antiretroviral (ARV) care clinics at the Wentworth provincial hospital and the Cator Manor community clinic in Durban, South Africa were recruited between September 2019 and June 2020. After completing written informed consent, women were administered study questionnaires on HIV status, reproductive and sexual history. Women who tested positive for HPV at enrolment were asked to return for treatment. During the visit biopsy or liquid based cytology (LBC) samples were taken for histology. Women returned after 1 year for repeat HPV testing.

### 2.3. Sample collection and testing

At the enrolment visit, after a pelvic examination by the nurse, a speculum aided cervical sample was taken by inserting the brush into the cervical os and rotating 4 times. Once removed the brush was placed into ThinPrep® PreservCyt solution®, (Hologic Inc, Marlborough, USA) and transported on the same day to the Clinical Medicine laboratory at the University of KwaZulu-Natal for HPV testing.

A pelvic exam was performed on women who returned for the treatment visit. In women whose squamous columnar junction (SCJ) was visible, biopsies were taken either from visible lesions or at 6 and 9 ^o^ clock in the absence of lesions. When the SCJ was not visible, a LBC sample was taken. These were couriered on the same day to an accredited pathology laboratory for histology and cytology analysis. Women were assessed and treated (if required) at the colposcopy unit at the King Edward VIII hospital if their SCJ was not visible or if they had been randomised to the arm without treatment but their histology results returned as a high grade squamous intraepithelial lesion (HSIL). Biopsy, LBC or LLETZ biopsy samples were sent to the pathology laboratory for analysis.

At the 1 year follow up visit, all women underwent a pelvic exam and a cervical brush was taken for HPV testing as described for the enrolment visit.

### 2.4. Detection of high -risk HPV (hrHPV) genotypes

Upon arrival at the laboratory, the collection brush was rotated several times into the Preservcyt® solution and removed by gently squeezing against the wall of the tube. The Cepheid Xpert® HPV assay, (Cepheid, Sunnyvale, USA), was performed. Approximately 1 ml of the inoculated Thinprep® solution was pipetted into the Xpert® HPV testing cartridge and placed into the automated GeneXpert instrument. The GeneXpert assay detects specific high-risk HPV genotypes which are grouped into 5 subgroups namely HPV 16; HPV P18_45; P3 (HPV 31/33/35/52/58); P4 (HPV 51/59); and P5 (HPV, 39/56/66/68). Post testing, positive samples and 10% of negative samples were stored in 1.5 ml aliquots at -80°C.

### 2.5. Histology and Cytology analysis

All histology and cytology samples were analysed at an accredited pathology laboratory in Durban, South Africa. A single pathologist was dedicated to the study. Standard slide preparation and staining was performed. Results were received indicating the cervical neoplasia grade.

### 2.6. Detection of cervico-vaginal bacteria

#### 2.6.1. DNA Extraction

The 1.5 ml aliquot of stored Thinprep® samples were thawed and centrifuged at 10000 rpm to sediment the cells and bacteria. The supernatant was discarded. DNA extraction followed the cultured cells protocol for small volumes using the Illustra Nucleon Genomics DNA extraction kit, (GE Healthcare life Sciences, little Chalfont, United Kingdom).

#### 2.6.2. PCR detection of cervico-vaginal bacteria

The primer sequences used in the real time PCR for the detection of the cervico-vaginal bacteria are detailed in Table 1.

All PCR reactions were carried out in the Quant Studio 5 PCR instrument (ThermoFisher Scientific, United States). The reaction mix contained 5 *μ*L PowerUp™ SYBR™ Master Mix (2x) (ThermoFisher Scientific, United Sates), 10 *μ*M of the forward and reverse primers, 1 *μ*L of template DNA and made up to a final volume of 10 *μ*L with sterile water. The cycling conditions used were: UDG activation stage for 2 minutes at 50° C, initial denaturation for 2 minutes at 95°C, denaturation for 15 seconds at 95°C, annealing 15 seconds at 52-60°C with extension for 1 minute at 72° C. To control for contamination and to assess efficiency of the PCR, a non-template (negative) control was included in all PCR runs.

### 2.7. Statistical analysis

The statistical data analysis was conducted in R Statistical computing software of the R Core Team, 2020, version 3.6.3. The results are presented in the form of descriptive and inferential statistics. The descriptive statistics of numerical measurements is summarized as the minimum, maximum, quartiles, interquartile range, means, standard deviation and the coefficient of variation. On the other hand, the categorical variables are described as counts and percentage frequencies. Depending on the distribution of the numerical variables between two independent groups, mean or median differences were assessed using either t-test or Wilcoxon, respectively. Multidimensional presentation of categorical variables used Likert plots. To determine the association between categorical variables, a Chi-Square Test was used and when the distribution of the cross tabulations contained an expected value of less than five, a Fisher's exact test was applied. All the predictor variables with the exception of BVAB 2 met the proportional odds assumption between negative (NILM), LSIL and HSIL, and were analysed with the application of ordinal regression analysis. BVAB 2 was analysed using multinomial regression with the negative as the referent. All the inferential statistical analysis tests were conducted at 5% levels of significance.

## 3. Results

### 3.1. Population characteristics

The prevalence of HPV in the study population was 56.4%.  Table 2 describes the demographic, behavioural and clinical factors across the HPV positive and negative women. According to the analysis, none of the variables investigated were statistically significant (p >0.05).

### 3.2. Prevalence of BV associated bacteria across HPV groups

The prevalence of *G. vaginalis* was 99.5% in the study population and therefore this microorganism was excluded from further analysis.

The prevalence of the other microorganisms were *Prevotella* spp (96.8%), *Lactobacillus* spp (81.1%), BVAB 1 (78.4%), *A vaginae* (74.2%), *S. sanguinegens* (68.9%), BVAB 3 (53.2%), and BVAB 2 (41.1%), Table 2.

The following organisms had a higher prevalence in the HPV positive group and all reached statistical significance: *A. vaginae* (90.7% vs 52.4%, p <0.001); BVAB1 (89.8% vs 63.4%, p <0.001); BVAB3 (64.8% vs 37.8%, p <0.001); *S. sanguinegens* (76.9% vs 58.5%, p =0.007) and *Lactobacillus* (87% vs 73.2%, p =0.016). BVAB 2 was detected in 43.5% of the HPV positive women when compared to the HPV negative group (37.8%), however this was not significant, p =0.428, Table 2. On the contrary, a higher prevalence of *Prevotella* spp was observed in HPV negative women (98.8%) when compared to HPV positive women (95.4%). However, this was not significant, p =0.238, Table 2.

### 3.3. Contributing factors in the severity of Cervical Intraepithelial Neoplasia (CIN)

For regression analysis on cervical neoplasia, low grade squamous intraepithelial lesion (LSIL) is defined as either having CIN grade 1 or abnormal squamous cells of undetermined significance (ASC-US). High grade squamous intraepithelial lesion (HSIL) is defined as CIN grade 2 or grade 3. BVAB 2 reduces the likelihood of having LSIL and HSIL compared to those who had a negative histology result, OR 0.25(0.09-0.66, p =0.005) and OR 0.49(0.17-1.41, p =0.184), respectively, Table 3. In the ordinal regression, detection of BVAB 3 is associated with a 2.08 (p =0.052) times greater likelihood of increasing CIN severity in the unadjusted model. This increases to 2.72 (p =0.079) times considering co-variables but does not reach statistical significance, Table 3. Detection of *A vaginae*, BVAB 1 and *S. sanguinegens* was associated with an increased odds for cervical neoplasia in the unadjusted analysis. This association is observed for both BVAB 1 (OR 1.51 : 0.42-5.55, p =0.525) and *S. sanguinegens* (OR 1.02 : 0.37-2.80, p =0.976) in the adjusted analysis. *Lactobacillus* is associated with a decreased likelihood of CIN for both the adjusted (OR 0.82 : 0.35-1.92, p =0.642) and unadjusted (OR 0.64 : 0.21-1.84, p =0.406) analysis, Table 3.

HPV infection regardless of hrHPV genotype was less likely to be associated with CIN however, after adjusting for confounding factors the odds increased to 1.77 times (p =0.518). Only HPV genotype P16 and HPV genotype group P3 were associated with increased odds of having cervical neoplasia. This was observed in the adjusted and unadjusted analysis, Table 3.

Increase in age per year was associated with a decreased likelihood for presenting with cervical neoplasia and was statistically significant in the unadjusted analysis, OR 0.95(0.91-1.00, p =0.041). Women who were gravida 1 [OR: 1.02(0.19-5.22, p =0.984) and OR: 1.15(0.19-6.90, p =0.878)] and those who had a regular partner [OR: 1.78(0.84-3.82, p =0.137) and OR: 2.11(0.85-5.34, p =0.110)] were also at an increased odds of having cervical neoplasia in both the adjusted and unadjusted model, respectively. The increasing number of lifetime sexual partners (p =0.412), increasing number of years on highly active antiretroviral therapy (HAART) (p =0.066) and increasing number of years since HIV diagnosis (p =0.019) were all associated with a decreased odds for CIN in the unadjusted analysis, Table 3.

### 3.4. Factors associated with HPV persistence and reinfection

In this study, persistence refers to the detection of the same HPV genotype/s at the one-year sampling as observed at enrolment. HPV reinfection is the detection of a different/additional HPV genotype at the 1 year sampling time point to that which was detected at enrolment. All of the BV associated bacteria analysed were detected more frequently in women with HPV persistence compared to those who tested negative at 1 year review: *A. vaginae* (94.6% vs 69.3%), BVAB 1 (91.9% vs 75.2%), BVAB 2 (51.4% vs 38.6%), BVAB 3 (64.9% vs 50.3%), *Lactobacillus* spp (83.8% vs 80.4%) and *S. sanguinegens* (81.1% vs 66%). *A vaginae* and BVAB 1 were statistically significant, p =0.002 and p =0.026, respectively. This increased frequency of detection is similarly observed for HPV reinfection however, only BVAB 3 reached statistical significance (p =0.010), Table 4.

All of the hrHPV genotypes and genotype groups tested were significantly associated with persistence of infection. While all of the hrHPV genotypes and genotype groups had an increased detection rate in the reinfection cohort, only HPV P16 was statistically significant, Table 4.

The median age of women with HPV persistence and reinfection was 39 years and 37 years, respectively. Gravida 0 and 1 was associated with both HPV persistence (p =0.017) and reinfection (p =0.143). Fewer women with gravida 2, 3 and 4 had HPV persistence and reinfection, Table 4. Increase by 1 in the number of lifetime sexual partners or sexual partners within the past 12 months, having a regular partner, number of years since HIV diagnosis and since HAART initiation were not significantly associated with either HPV persistence or reinfection, Table 4.

## 4. Discussion

In this study, we looked at PCR detection of specific vaginal bacteria associated with BV in HIV positive women with and without a co-infection with HPV. Previous studies on South African HIV positive cohorts have reported a higher prevalence of BV in women co-infected with HIV and an increased acquisition and persistence of HPV [18, 19]. In keeping with previous reports, *A. vaginae*, BVAB 1, BVAB 2, BVAB 3, *S. sanguinegens* and *Lactobacillus* spp were detected more frequently in HPV positive patients.

We show a significant association between the detection of *Lactobacillus* spp and HPV infection. This is in keeping with a report from Beijing that found *L. gasseri* was significantly associated with detection of HPV [20]. Our method of detection used a genus specific primer, use of species-specific primers or microbiome sequencing would determine the *Lactobacillus* species responsible for the association observed.

HIV positive women co-infected with HPV are 2.3 times more likely to have BV and BVAB1, BVAB 2 and BVAB 3 are indicators of BV in these women [21, 22]. We report a statistically significant association of BVAB 1 and 3 and an increased detection of BVAB 2 in women co-infected with HPV.

Despite an observation of *A. vaginae* and *G. vaginalis* in over 80% of Mexican women, there was no association to HPV infection; authors concluded that these bacteria might be part of the normal microbiome [23]. There are conflicting reports in the association of *Sneathia* species in HPV infection of Chinese women [24, 25]. Onywera et al., (2019) reported an increased abundance of both *Sneathia* and *A. vaginae* in hrHPV infected women who were HIV negative from South Africa [10]. In this study, *G. vaginalis* was observed in all but one sample and was not included in the analysis, however, the pathogenicity of *A. vaginae* and *S. sanguinegens* is highlighted by the significant associations with hrHPV infection.

Studies indicate apart from hrHPV, there may be contributing factors in the development and progress of CIN. Therefore, HPV may not be the sole factor for progression of cervical neoplasia severity [26]. This is in keeping with our observation as confounding factors increased the odds for increasing CIN. In Norwegian women with histologically confirmed CIN2 or higher, there was a higher oncogenic potential of HPV 16 and HPV33 [27]. We report increased odds for increasing CIN severity for HPV 16 and HPV genotype group3, the latter includes HPV 33 and three other HPV genotypes consistent with the Norwegian study. This study was limited by a grouped HPV genotype detection; therefore, the genotype driving the association is unknown.

In Brazilian women, BV was associated with an increased odds of having CIN2+. This was independent of the inflammatory response observed [28]. In women histologically confirmed as CIN2, *Atopobium* species, BVAB1, *Sneathia*, *Megaspheara* and *Prevotella* were predictors of non-regression at 12 months using LefSe analysis [29]. Another study on a cohort of Hispanic women using DeSeq2 for the identification of differential taxa, reported an enrichment of *Sneathia* spp. in the groups with low and high grade dysplasia [30]. In support of these finding we report an increased odds for increasing CIN severity when the following BV associated bacteria were detected by PCR: *A. vaginae*, BVAB 1, BVAB 3 and *S. sanguinegens*. The latter 3 bacterial types maintained increased odds after adjusting for confounding factors. BVAB 3 was borderline significant in the unadjusted analysis, however, in the adjusted analysis significance disappears.

An abundance of *A. vaginae* was found to increase the risk of CIN in women from Korea, this effect was increased in the presence of hrHPV [31]. In our study we did not estimate abundance of bacterial types detected however, detection of *A vaginae* was associated with an increased odds for cervical neoplasia in women with hrHPV. This association disappears when adjusting for confounding factors which include presence of hrHPV. This is a possible indication that *A vaginae* is not an independent but rather a cofactor in cervical neoplasia. Alternately, this picture may change if we focus on abundance of *A. vaginae* in relation to CIN severity.

Different *Lactobacillus* species have an association to increased CIN relative to either abundance or co-infection with hrHPV [32, 33]. Using the ordinal regression model we did not observe such an association. We report a lack of association of *Lactobacillus* species to increasing CIN. The limitation of our study is the use of a genus specific primer set. The different species of *Lactobacillus* present in this cohort is unknown. Identification and analysis at a species level may change the observation reported.

A global data review reported a 3 fold increased incidence of cervical lesions in HIV positive women [34]. In Taiwan there is an increased risk of cervical neoplasia in women infected with HIV and the use of HAART significantly lowered this risk [35]. In our HIV positive cohort, the increase per year on HAART was associated with a lesser likelihood of cervical neoplasia. The increase per year since HIV diagnosis had a significantly lower odds of detection of CIN. This could be explained by adherence to treatment as the women recruited in this study are those seeking HAART treatment. However, in the adjusted analysis HAART had a 1.09 times increased association to CIN, this was not significant and is likely driven by confounding factors.

Guo et al. reported similar BV detection rates at baseline between the HPV persistent group and those that cleared the infection. They also found a significantly higher number of women had BV at the end of the study in the HPV persistent group [36]. We report an increased level of detection of all the BV associated bacterial types tested in the group with HPV persistence. This infers that the condition of BV and the bacteria associated with BV contributes to HPV persistence. This study did not determine bacterial detection at the end of the study. We report a significant association between *A. vaginae* and BVAB 1 in HPV persistence. This is in keeping with a previous report that indicate an abundance of *Atopobium* spp in HPV persistence [37].

Dareng et al. reported that *Lactobacillus* dominance in HIV negative women had a lower association with persistent hrHPV infection, while in HIV positive women a *Lactobacillus* dominant vaginal microbiome was associated with increased odds of persistent hrHPV infection [38]. Of the 37 women in our study that had persistent HPV infection, 83.8% had detectable levels of *Lactobacillus* spp. This was marginally higher than the non-persistent group (80.4%). Determination of bacterial dominance may change this association.

Similar to the data on persistence, we show that all of the BV associated bacteria detected had a higher frequency of detection in the women with incident (reinfection) hrHPV infection. Only detection of BVAB 3 was significantly associated with incident hrHPV infection at the 1-year visit. Another study found that BV had an increased odds for prevalent and incident HPV as well as delayed clearance of HPV infection [39].

All of the hrHPV genotypes or genotype groups in this study were detected at a significantly higher frequency in the group with HPV persistence. However, only HPV 16 was significantly associated with reinfection at the 1-year visit. Of the behavioural and biological factors, gravida had a significant association to HPV persistence. Interestingly there were a greater number of women who were gravida <2 in the groups with HPV persistence and reinfection. There was a greater number of women who were gravida ≥2 in the groups without HPV persistence and reinfection. Increasing gravida has been previously reported to be associated with increasing HPV infection [40]. Yet another study found that HPV infection decreased at higher number of pregnancies [41].

In this study we have shown that BV associated bacteria are detected more frequently in women with a coinfection with hrHPV. This is in keeping with previous literature reporting the association of either BV or BV associated bacteria with HPV infection [24, 39]. Significant association to prevalent hrHPV infection in this study is described for *A. vaginae*, BVAB 1, BVAB 3, *S. sanguinegens* and *Lactobacillus* spp. BV is prevalent in HIV positive South African women and specifically BVAB 1, BVAB 2 and BVAB 3 are indicative of BV in HIV positive women [19, 22]. Therefore, women living with HIV are at a higher risk of HPV infection due to their susceptibility to BV. BVAB 2 although detected more frequently in HPV infection, showed no significant association to prevalent HPV, HPV persistence or reinfection. HPV persistence is accepted as fundamental to the development of cervical neoplasia [42]. BV associated bacteria of interest in this study were also detected more frequently in HPV persistence and HPV reinfection. Specifically, *A. vaginae* and BVAB 1 were statistically significant in HPV persistence while BVAB 3 was significantly associated with HPV reinfection. Of the BV associated bacteria detected in this study, BVAB 2 and *Lactobacillus* spp were not associated with increasing CIN severity. However, BVAB 1, BVAB 3, *A. vaginae* and *S. sanguinegens* had an increased odds for increasing CIN severity in the unadjusted analysis. BVAB 3 was borderline significant. *S. sanguinegens* and BVAB 1 retained an increased association for increasing CIN severity in the adjusted model. While infection with any hrHPV has a decreased odds for CIN severity in the unadjusted analysis, contributing20 effects of co-variables result in an increased association in the adjusted analysis, However, HPV 16 and HPV genotype group P3 are independently associated with increased odds for CIN severity. Gravida 1 is also independently associated with CIN severity. However, neither HPV genotypes nor gravida are significant.

## 5. Conclusion

We conclude that specific BV associated bacteria increases the risk of HPV acquisition in HIV positive South African women and the risk of cervical neoplasia is heightened by the association of these bacteria to HPV persistence. BVAB 1 and BVAB 3 may be biomarkers for increased risk of cervical neoplasia as they have been shown to be statistically significant in prevalent HPV infection, statistically significant in HPV persistence and reinfection, respectively, and both have an independent increased odds for cervical neoplasia in the adjusted and unadjusted models. BVAB 3 reaching borderline significance in the latter. We recommend point of care BV testing in high risk women especially those attending ARV clinics and closer monitoring of women positive for BV are required. Additionally, the pathogenesis of these specific bacteria in HPV infection and CIN progression requires further investigation.

## Figures and Tables

**Table 1:** Primer sequences for real time PCR of cervico-vaginal bacteria.

Microorganism	Sequence (5^'-^3′)	Amplicon size	Reference
*Atopobium vaginae* Forward Reverse	TAGGTCAGGAGTTAAATCTG TCATGGCCCAGAAGACCGCC	155 bp	[14]
BVAB1 Forward Reverse	GGAGTGTAGGCGGCACTA CTCTCCGATACTCCAGCTCTA	90 bp	[14]
BVAB2 Forward Reverse	TTAACCTTGGGGTTCATTACAA GAATACTTATTGTGTTAACTGCGC	260 bp	[14]
BVAB3 Forward Reverse	CATTTAGTTGGGCACTCAGGC ACATTTGGGGATTTGCTTCGCC	160 bp	[14]
*Sneathia sanguineges* Forward Reverse	AATTATTGGGCTTAAAGGGCATC AGTACTCTAGTTATACAGTTTTGTAG	102 bp	[14]
*Prevotella* spp. Forward Reverse	CCAGCCAAGTAGCGTGCA TGGACCTTCCGTATTACCGC	160-170 bp	[15]
*Lactobacillus* spp. Forward Reverse	TACATCCCAACTCCAGAACG AAGCAACAGTACCACGACC	90 bp	[16]
*Gardnerella vaginalis* Forward Reverse	TTACTGGTGTATCACTGTAA CCGTCACAGGCTGAACAGT	330 bp	[14, 17]

**Table 2:** Population characteristics and the association of BV associated bacteria in participants co-infected with HPV at enrolment.

	HPV Negative (N =82)	HPV Positive (N =108)	*p*-value	Overall (N =190)
A. vaginae			p <0.001	
Negative	39 (47.6%)	10 (9.3%)		49 (25.8%)
Positive	43 (52.4%)	98 (90.7%)		141 (74.2%)
BVAB 1			p <0.001	
Negative	30 (36.6%)	11 (10.2%)		41 (21.6%)
Positive	52 (63.4%)	97 (89.8%)		149 (78.4%)
BVAB 2			p =0.428	
Negative	51 (62.2%)	61 (56.5%)		112 (58.9%)
Positive	31 (37.8%)	47 (43.5%)		78 (41.1%)
BVAB 3			p <0.001	
Negative	51 (62.2%)	38 (35.2%)		89 (46.8%)
Positive	31 (37.8%)	70 (64.8%)		101 (53.2%)
Lactobacillus spp			p =0.016	
Negative	22 (26.8%)	14 (13.0%)		36 (18.9%)
Positive	60 (73.2%)	94 (87.0%)		154 (81.1%)
Prevotella spp			p =0.238	
Negative	1 (1.2%)	5 (4.6%)		6 (3.2%)
Positive	81 (98.8%)	103 (95.4%)		184 (96.8%)
S. sanguinegens			p =0.007	
Negative	34 (41.5%)	25 (23.1%)		59 (31.1%)
Positive	48 (58.5%)	83 (76.9%)		131 (68.9%)
Age			p =0.392	
Mean ± SD(CV%)	39.4 ± 8.17(20.7)	40.3 ± 8.42(20.9)		39.9 ± 8.31(20.8)
Median(Q1-Q3)	39.0(33.0-45.0)	42.0(33.0-46.0)		40.5(33.0-46.0)
Min-Max	25.0-54.0	25.0-54.0		25.0-54.0
Gravida			p =0.533	
0	5 (6.1%)	7 (6.5%)		12 (6.3%)
1	19 (23.2%)	38 (35.2%)		57 (30.0%)
2	25 (30.5%)	31 (28.7%)		56 (29.5%)
3	16 (19.5%)	16 (14.8%)		32 (16.8%)
4	10 (12.2%)	8 (7.4%)		18 (9.5%)
5+	7 (8.5%)	8 (7.4%)		15 (7.9%)
No of lifetime sexual partners			p =0.314	
Mean ± SD(CV%)	3.00 ± 1.36(45.2)	3.57 ± 2.32(65.1)		3.35 ± 2.02(60.2)
Median(Q1-Q3)	3.00(2.00-4.00)	3.00(2.00-5.00)		3.00(2.00-4.00)
Min-Max	1.00-6.00	1.00-10.0		1.00-10.0
No of sex partners in past 12 months			p =0.804	
Mean ± SD(CV%)	0.859 ± 0.418(48.7)	0.883 ± 0.676(76.5)		0.873 ± 0.578(66.2)
Median(Q1-Q3)	1.00(1.00-1.00)	1.00(1.00-1.00)		1.00(1.00-1.00)
Min-Max	0-2.00	0-6.00		0-6.00
Having a regular partner			p =0.211	
No	24 (29.3%)	41 (38.0%)		65 (34.2%)
Yes	58 (70.7%)	67 (62.0%)		125 (65.8%)
Time on ART(years)			p =0.317	
Mean ± SD(CV%)	7.24 ± 4.78(66.0)	6.59 ± 4.86(73.7)		6.88 ± 4.82(70.1)
Median(Q1-Q3)	6.00(4.00-11.0)	5.50(2.00-10.0)		6.00(3.00-10.0)
Min-Max	0-24.0	0-18.0		0-24.0
No of years since HIV diagnosis			p =0.530	
Mean ± SD(CV%)	8.30 ± 5.27(63.5)	7.79 ± 5.52(70.9)		8.01 ± 5.41(67.5)
Median(Q1-Q3)	8.00(4.00-12.0)	8.00(2.75-12.0)		8.00(3.00-12.0)
Min-Max	0-26.0	0-23.0		0-26.0

**Table 3:** Factors associated with the severity of Cervical Intraepithelial Neoplasia (CIN).

	UNADJUSTED	ADJUSTED
	OR(95% CI,p.value)	OR(95% CI,p.value)
*A vaginae* Positive	1.09(0.45-2.61, p =0.850)	0.39(0.09-1.71, p =0.215)
BVAB 1 Positive	1.34(0.51-3.51, p =0.551)	1.51(0.42-5.55, p =0.525)
BVAB 3 Positive	2.08(1.00-4.40, p =0.052)	2.72(0.90-8.55, p =0.079)
*Lactobacillus* spp Positive	0.82(0.35-1.92, p =0.642)	0.64(0.21-1.84, p =0.406)
*S. sanguinegens* Positive	1.18(0.54-2.59, p =0.679)	1.02(0.37-2.80, p =0.976)
HPV Infection	0.94(0.37-2.39, p =0.901)	1.77(0.32-10.20, p =0.518)
HPV P16 Positive	1.98(0.70-5.76, p =0.202)	1.17(0.36-3.87, p =0.796)
HPV P18_45 Positive	0.79(0.32-1.91, p =0.599)	0.51(0.16-1.61, p =0.251)
HPV group P3 Positive	1.64(0.80-3.38, p =0.180)	1.20(0.43-3.34, p =0.730)
HPV group P4 Positive	0.55(0.21-1.39, p =0.206)	0.34(0.10-1.16, p =0.087)
HPV group P5 Positive	0.95(0.43-2.10, p =0.893)	0.80(0.29-2.11, p =0.647)
Age	0.95(0.91-1.00, p =0.041)	0.97(0.91-1.03, p =0.364)
Gravida1	1.02(0.19-5.22, p =0.984)	1.15(0.19-6.90, p =0.878)
Gravida2	0.65(0.12-3.33, p =0.599)	0.84(0.14-5.23, p =0.850)
Gravida3	0.30(0.05-1.82, p =0.190)	0.40(0.05-3.10, p =0.373)
Gravida4	0.34(0.04-2.46, p =0.284)	0.43(0.04-5.05, p =0.494)
Gravida5+	0.27(0.04-1.87, p =0.184)	0.35(0.03-3.79, p =0.382)
No. of lifetime sexual partners	0.93(0.79-1.10, p =0.412)	0.87(0.71-1.06, p =0.161)
Having a regular partner	1.78(0.84-3.82, p =0.137)	2.11(0.85-5.34, p =0.110)
No. of sex partners in past 12 months	1.09(0.66-1.87, p =0.729)	0.76(0.40-1.48, p =0.389)
Time on HAART (years)	0.93(0.86-1.00, p =0.066)	1.09(0.87-1.39, p =0.447)
No. of years since HIV diagnosis	0.92(0.85-0.99, p =0.019)	0.87(0.70-1.08, p =0.216)
	**LSIL (UNADJUSTED)**	**HSIL (UNADJUSTED)**
∗BVAB 2 Positive	0.25(0.09-0.66, p =0.005)	0.49(0.17-1.41, p =0.184)

∗BVAB 2 is analysed as a multinomial regression with the referent being the group with normal histology (NILM) and the rest are based on the ordinal logistic regression.

**Table 4:** Factors associated with HPV persistence and reinfection in HIV positive women.

	HPV Persistence		p-value	HPV Reinfection			Overall (N =190)
	No (N =153)	Yes (N =37)	p-value	No (N =179)	Yes (N =11)	p-value	Overall (N =190)
A. vaginae			p =0.002			p =0.069	
Negative	47 (30.7%)	2 (5.4%)		49 (27.4%)	0 (0.0%)		49 (25.8%)
Positive	106 (69.3%)	35 (94.6%)		130 (72.6%)	11 (100.0%)		141 (74.2%)
BVAB 1			p =0.026			p =0.125	
Negative	38 (24.8%)	3 (8.1%)		41 (22.9%)	0 (0.0%)		41 (21.6%)
Positive	115 (75.2%)	34 (91.9%)		138 (77.1%)	11 (100.0%)		149 (78.4%)
BVAB 2			p =0.156			p =0.762	
Negative	94 (61.4%)	18 (48.6%)		106 (59.2%)	6 (54.5%)		112 (58.9%)
Positive	59 (38.6%)	19 (51.4%)		73 (40.8%)	5 (45.5%)		78 (41.1%)
BVAB 3			p =0.112			p =0.010	
Negative	76 (49.7%)	13 (35.1%)		88 (49.2%)	1 (9.1%)		89 (46.8%)
Positive	77 (50.3%)	24 (64.9%)		91 (50.8%)	10 (90.9%)		101 (53.2%)
Lactobacillus spp			p =0.637			p =0.693	
Negative	30 (19.6%)	6 (16.2%)		35 (19.6%)	1 (9.1%)		36 (18.9%)
Positive	123 (80.4%)	31 (83.8%)		144 (80.4%)	10 (90.9%)		154 (81.1%)
S. sanguinegens			p =0.075			p =0.177	
Negative	52 (34.0%)	7 (18.9%)		58 (32.4%)	1 (9.1%)		59 (31.1%)
Positive	101 (66.0%)	30 (81.1%)		121 (67.6%)	10 (90.9%)		131 (68.9%)
HPV P16			p =0.021			p =0.031	
Negative	139 (90.8%)	28 (75.7%)		160 (89.4%)	7 (63.6%)		167 (87.9%)
Positive	14 (9.2%)	9 (24.3%)		19 (10.6%)	4 (36.4%)		23 (12.1%)
HPV P18_45			p =0.013			p =0.193	
Negative	136 (88.9%)	27 (73.0%)		155 (86.6%)	8 (72.7%)		163 (85.8%)
Positive	17 (11.1%)	10 (27.0%)		24 (13.4%)	3 (27.3%)		27 (14.2%)
HPV group P3			p <0.001			p =0.190	
Negative	113 (73.9%)	12 (32.4%)		120 (67.0%)	5 (45.5%)		125 (65.8%)
Positive	40 (26.1%)	25 (67.6%)		59 (33.0%)	6 (54.5%)		65 (34.2%)
HPV group P4			p =0.003			p =0.120	
Negative	141 (92.2%)	27 (73.0%)		160 (89.4%)	8 (72.7%)		168 (88.4%)
Positive	12 (7.8%)	10 (27.0%)		19 (10.6%)	3 (27.3%)		22 (11.6%)
HPV group P5			p <0.001			p =0.235	
Negative	134 (87.6%)	18 (48.6%)		145 (81.0%)	7 (63.6%)		152 (80.0%)
Positive	19 (12.4%)	19 (51.4%)		34 (19.0%)	4 (36.4%)		38 (20.0%)
Age			p =0.379			p =0.099	
Mean ± SD(CV%)	40.2 ± 8.15 (20.3)	38.9 ± 8.96 (23.0)		40.2 ± 8.29(20.6)	35.6 ± 7.65(21.5)		39.9 ± 8.31(20.8)
Median(Q1-Q3)	41.0(33.0-46.0)	39.0(31.0-45.0)		41.0(33.0-46.5)	37.0(28.5-43.0)		40.5(33.0-46.0)
Min-Max	24.0-54.0	25.0-54.0		24.0-54.0	25.0-44.0		24.0-54.0
Gravida			p =0.017			p =0.143	
0	8 (5.2%)	4 (10.8%)		10 (5.6%)	2 (18.2%)		12 (6.3%)
1	39 (25.5%)	18 (48.6%)		51 (28.5%)	6 (54.5%)		57 (30.0%)
2	46 (30.1%)	10 (27.0%)		55 (30.7%)	1 (9.1%)		56 (29.5%)
3	29 (19.0%)	3 (8.1%)		31 (17.3%)	1 (9.1%)		32 (16.8%)
4	16 (10.5%)	2 (5.4%)		17 (9.5%)	1 (9.1%)		18 (9.5%)
5+	15 (9.8%)	0 (0.0%)		15 (8.4%)	0 (0.0%)		15 (7.9%)
No. of lifetime sexual partners			p =0.355			p =0.288	
Mean ± SD(CV%)	3.38 ± 1.95(57.6)	3.21 ± 2.27(70.7)		3.29 ± 1.97(60.0)	4.09 ± 2.51(61.3)		3.35 ± 2.02(60.2)
Median(Q1-Q3)	3.00(2.00-4.00)	3.00(1.25-4.00)		3.00(2.00-4.00)	4.00(2.50-5.00)		3.00(2.00-4.00)
Min-Max	1.00-10.0	1.00-10.0		1.00-10.0	1.00-10.0		1.00-10.0
No of sex partners in past 12 months			p =0.441			p =0.829	
Mean ± SD(CV%)	0.890 ± 0.600(67.4)	0.800 ± 0.473(59.1)		0.876 ± 0.588(67.1)	0.818 ± 0.405(49.4)		0.873 ± 0.578(66.2)
Median(Q1-Q3)	1.00(1.00-1.00)	1.00(1.00-1.00)		1.00(1.00-1.00)	1.00(1.00-1.00)		1.00(1.00-1.00)
Min-Max	0-6.00	0-2.00		0-6.00	0-1.00		0-6.00
Having a regular partner			p =0.197			p =0.752	
No	49 (32.0%)	16 (43.2%)		62 (34.6%)	3 (27.3%)		65 (34.2%)
Yes	104 (68.0%)	21 (56.8%)		117 (65.4%)	8 (72.7%)		125 (65.8%)
Time on HAART (years)			p =0.425			p =0.689	
Mean ± SD(CV%)	6.72 ± 4.74(70.5)	7.51 ± 5.16(68.7)		6.90 ± 4.80(69.5)	6.45 ± 5.41(83.8)		6.88 ± 4.82(70.1)
Median(Q1-Q3)	6.00(3.00-10.0)	8.00(2.00-11.0)		6.00(3.00-10.0)	7.00(1.50-10.0)		6.00(3.00-10.0)
Min-Max	0-24.0	0-18.0		0-24.0	0-15.0		0-24.0
No. of years since HIV diagnosis			p =0.830			p =0.417	
Mean ± SD(CV%)	8.02 ± 5.31(66.3)	7.97 ± 5.86(73.5)		8.09 ± 5.40(66.7)	6.73 ± 5.66(84.1)		8.01 ± 5.41(67.5)
Median(Q1-Q3)	8.00(3.00-12.0)	8.00(2.00-11.0)		8.00(3.00-12.0)	7.00(1.50-10.5)		8.00(3.00-12.0)
Min-Max	0-26.0	0-23.0		0-26.0	0-16.0		0-26.0

## Data Availability

The corresponding author can be contacted for data requests.
